# Understanding disease burden, challenges in current treatment strategies and call for action for management of severe asthma in Asia: a position statement from Asian respiratory experts

**DOI:** 10.3389/falgy.2026.1738118

**Published:** 2026-02-26

**Authors:** Mariko Siyue Koh, Ken Ka Pang Chan, Koichi Fukunaga, Sang-Heon Kim, Le Thi Tuyet Lan, Azza Omar, Thitiwat Sriprasart, Deepak Talwar, Min Zhang, Shih-Lung Cheng

**Affiliations:** 1 Respiratory and Critical Care Medicine, Singapore General Hospital, Singapore, Singapore; 2 Duke- National University Medical School, Singapore, Singapore; 3 Department of Medicine & Therapeutics, Faculty of Medicine, Chinese University of Hong Kong, Shatin, New Territories, Hong Kong SAR, China; 4 Li Ka Shing Institute of Health Sciences, Faculty of Medicine, Chinese University of Hong Kong, Shatin, New Territories, Hong Kong SAR, China; 5 Division of Pulmonary Medicine, Department of Medicine, Keio University School of Medicine, Tokyo, Japan; 6 Department of Internal Medicine, Hanyang University College of Medicine, Seoul, Republic of Korea; 7 Respiratory Care Centre, University Medical Centre Ho Chi Minh City, Ho Chi Minh City, Vietnam; 8 Respiratory Unit, Medical Department Hospital Raja Perempuan Zainab II, Kota Bharu, Kelantan, Malaysia; 9 Division of Pulmonary and Critical Care, Department of Medicine, Faculty of Medicine, Chulalongkorn University and King Chulalongkorn Memorial Hospital, Thai Red Cross Society, Bangkok, Thailand; 10 Metro Centre for Respiratory Diseases, Noida, India; 11 Department of Respiratory and Critical Care Medicine, Shanghai General Hospital, Shanghai Jiao Tong University School of Medicine, Shanghai, China; 12 Department of Pulmonary Medicine, Far Eastern Memorial Hospital, New Taipei City, Taiwan; 13 Department of Chemical Engineering and Materials Science, Yuan Ze University, Taoyuan, Taiwan

**Keywords:** biologic agents, clinical remission, severe asthma, severe asthma burden, severe asthma in Asia

## Abstract

In the Asia-Pacific region, there is limited data on the full extent of the burden of severe asthma, as well as a lack of clear guidance on improving care standards. Genetic, environmental, behavioural, policy and funding factors in -Asia-Pacific region differ from other parts of the world. To address these gaps, a panel of Asian experts conducted a comprehensive review of the existing literature in the region, aiming to understand the burden of severe asthma and provide key recommendations and actionable steps to improve its management across Asia. Experts identified several key challenges in managing SA, which include inadequate expertise, delayed patient identification or referral, limited access to advanced diagnostics and treatments, suboptimal adherence, and insufficient government support and funding, underlining the need for a more comprehensive approach to SA management. Experts also proposed that the Asia-Pacific region definition of clinical remission in SA should include elimination of exacerbation and use of oral corticosteroids, good symptom control, and inclusion of lung function criteria, particularly in light of the evolving treatment landscape. The experts concluded that while restoring normal lung function may be unrealistic for most patients with severe asthma and remodelled airways, striving for optimal individual lung function or maintaining stability may be a more attainable goal. Several key points and actionable recommendations were proposed to help reduce the overall burden of severe asthma in Asia.

## 1 Introduction

Severe asthma (SA) is generally defined as asthma that remains uncontrolled despite adherence to maximal optimized inhaled therapy and treatment of contributory factors, or that worsens when high-dose inhaled treatment is reduced. Slight variations in this definition exist depending on the country or society providing it (1–5). SA is estimated to affect approximately 3.7% of all asthma patients globally (1), although prevalence can range from 1.8% to 38% (6). They account for a disproportionate share of healthcare utilization and costs (7).

Uncontrolled asthma is characterised by having at least one of the following criteria: poor symptom control, indicated by an Asthma Control Questionnaire (ACQ) consistently ≥1.5 or an Asthma Control Test (ACT) score of less than 20, or when asthma is classified as “not well controlled” according to National Asthma Education and Prevention Program (NAEPP)/GINA guidelines; and/or frequent severe exacerbations are defined as two or more instances of systemic corticosteroid bursts, each lasting at least three days, within the past year; and/or serious exacerbations, which involve at least one instance of hospitalization, an Intensive Care Unit (ICU) stay, or the need for mechanical ventilation in the previous year; and//or airflow limitation, where, after withholding an appropriate bronchodilator, the Forced Expiratory Volume in 1 s (FEV_1_) is less than 80% of the predicted value, accompanied by a reduced FEV_1_/ Forced Vital Capacity (FVC) ratio that falls below the lower limit of normal (<0.70) (2, 8, 9).

In the Asia-Pacific region, asthma-related risk factors are highly prevalent, particularly in the developing countries (10). Factors contributing to the rising number of asthma cases and their severity include environmental exposures like urbanization, air pollution, occupational and biomass fuel exposure, indoor pollutants (e.g., smoking, vaping, burning incense), and allergens such as dust mites, pet dander, and fungi (10). Additionally, healthcare resource limitations, especially in low- and middle-income countries (LMICs) with limited access to medications and severe asthma centres, exacerbate the issue. Low health literacy and awareness about SA, limited funding for SA, increasing atmospheric temperature, increasing incidence of obesity, and beliefs such as a preference for traditional remedies further complicate the situation (10–13).

The 2025 GINA recommendations outline a stepwise approach to asthma treatment, tailored to the patient's symptoms and medication requirements (5, 14). Despite significant advances in asthma care, many patients with SA continue to experience uncontrolled symptoms, frequent exacerbations, and reduced quality of life. In the Asia-Pacific region, there is limited data on the full extent of the burden of severe asthma, as well as a lack of clear guidance on improving care standards. Additionally, the clinical features and underlying causes of SA in this region may differ from those in other parts of the world (10–13).

These factors highlight the need for region-specific recommendations for optimal management of SA. To address these gaps, a panel of Asian experts conducted a comprehensive review of the existing literature in the region, aiming to understand the burden of severe asthma and provide key recommendations and actionable steps to improve its management across Asia.

## 2 Methods

### 2.1 Literature search and review

A comprehensive literature search was conducted on the PubMed databases to identify relevant studies for the identification of literature based on various themes of the position statements and objectives of the study. The methodology included search terms used across databases, countries/regions covered in the review, timeframe for the literature search, outcomes and populations considered and clear definition of who the authors would be, how they were selected, and their roles in the review process. The Position Statement attempts to:

- Review the recent evidence in Asia on the following topics:
  (i) Disease burden
  (ii) Factors associated with SA and poor outcomes in patients with SA
  (iii) Management of SA in Asia-Pacific and challenges faced
  (iv) Presence and proportion of “Asian phenotypes”
  (v) Clinical remission in SA from an Asian perspective
  (vi) Recommendations and call for actions in improving the standard of severe asthma care in Asia-Pacific via the adoption of a “treat-to-target” approach (clinical remission)

The search terms used included: severe asthma-related terms, burden-related terms, remission terms, and Asia Pacific-related territories terms. Additional relevant literature was identified using grey literature search.

The search was restricted to studies conducted in human populations, full-text articles, and the English language; however, no time period restriction was applied.

### 2.2 Participants

A diverse group of key opinion leaders in the field of Pulmonology from several regions across Asia collaborated over a series of virtual monthly meetings between September 2024 and March 2025 to define the topics for the position statement, identify and review existing literature, develop position statements, and write, review, and approve the statements.

### 2.3 Survey

A short survey was also conducted to gather experts' opinions on topics such as the potential definition of clinical remission from an Asian perspective, as well as the availability and accessibility of biologics in their respective countries. The survey was administered online in December 2024.

## 3 Results

**Figure 1:**
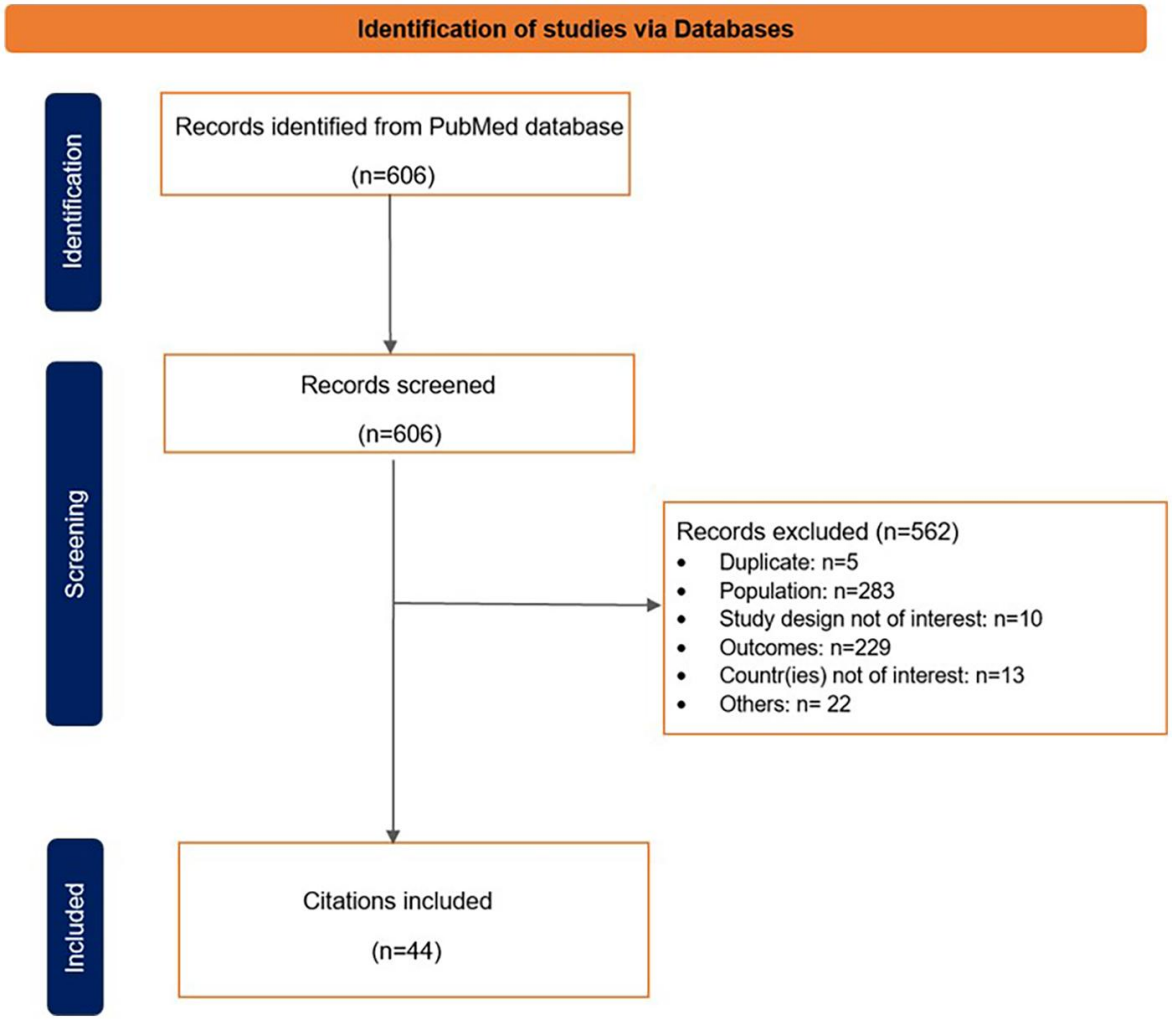
PRISMA flowchart to summarize the selection process of the eligible studies.

An overview of the results of the literature search is summarized in Figure 1.

### 3.1 Disease burden and challenges in the management of severe asthma in Asia

#### 3.1.1 Prevalence of severe asthma in Asia

The ERS/ATS guidelines estimate that severe asthma affects between 5% and 10% of the total asthma population globally (2). Studies conducted in Asian countries reveal a prevalence of SA to be as high as 50%, higher than the global prevalence. Rabe et al. reported that while the global SA prevalence was approximately 27% of all asthma cases, in Asia, 31% of all cases were SA. Country-wise SA prevalence was reported to be 1% in Thailand, 2.7% in Malaysia, and 7%–11% in Vietnam and Singapore. Notably, India had a lower overall prevalence of asthma (2.75%); however, 50% of these cases were SA (15). According to the Epidemiological Survey conducted in China (CARE), the SA prevalence was reported to be 7.1%, which is similar to another study reporting 7.2% (4, 16). Some regions in Asia have shown gradually increasing trends, particularly Japan, Korea, China, and India. In Japan, the prevalence of SA fluctuated over time, varying from 5.6% in 2013 (17), to 7.8% in 2015 (18), and 4.6% in 2017 (17). In Korea, the prevalence of SA ranged from 2.9% to 6.1% (19, 20). Meanwhile, a Taiwan study reported a 6.2% prevalence of SA (21). This trend has steadily increased over the years in Korea, with the proportion of patients with SA rising from 3.5% in 2002 to 6.1% in 2015 of the total asthma population (20).

Rapid urbanisation, indoor biomass fuels, and outdoor air pollution, lifestyle factors such as obesity and increased prevalence of smoking, ageing population, nutritional factors, and genetic factors were identified to be the potential contributors to the increasing prevalence of SA in Asia (22, 23).

**Table:** 

Severe asthma seems to affect a larger proportion of asthma patients in Asia as compared with the global data.
Risk factors such as rapid urbanisation, exposure to outdoor and indoor pollution, and genetic factors could be contributing to the higher rate of SA

### 3.2 Burden of severe asthma in Asia

#### 3.2.1 Healthcare resource utilisation

##### 3.2.1.1 Hospitalization

Severe asthma is associated with an increase in the rate of hospitalization. In a recent study conducted by Yang et al, SA was associated with 3.9–4.4-fold higher rates as compared with the average of the overall population in China. These hospitalizations resulted in a spending of Chinese Yuan (CNY) 6,782 (USD 937.48) per patient per year, with a 57% hospitalization rate (16). In a study by Inoue et al. (2009–2015) using anonymized data from the Japan Medical Data Center, SA was linked to significantly higher rates of hospitalization, outpatient visits, and prescriptions compared to non-severe asthma (IRRs: 1.60, 1.43, and 1.24, respectively) (24). Another Japanese study reported a 6.2% hospitalization rate among patients with severe controlled asthma (SCA), compared to 3.0% hospitalization rate among those with mild to moderate asthma. The average annual length of hospitalization was 4.51 days (SD 19.50) for SUA, (severe uncontrolled asthma), 1.78 days (SD 12.59) for SCA and 0.92 days (SD 12.42) for mild to moderate asthma (18). The SUC definition included that poor symptoms control (ACT less than 19), frequent exacerbations and OCS dependence under optimal therapy (GINA step 5 therapy). A study conducted in Thailand reported a 45.2% rate of hospitalization in severe persistent asthma patients as compared with 9.1% of patients with intermittent asthma in the previous year (25). A 2015 cross-sectional study found that severe asthma accounted for 43.9% of hospital admissions for asthma within the study population in Singapore (26). In South Korea, the annual hospitalization rate for SA was 2.1 per year in 2014, which was significantly higher than that for mild and moderate asthma (27).

#### 3.2.2 Economic burden

##### 3.2.2.1 Medical costs

Kimura et al. conducted a study using a Japanese national administrative database and reported that the median annual costs for SA increased from USD 9,543 in 2013 to USD 13,284 in 2019 (17). Another study reported that the mean all-cause medical costs increased from USD 3,404.6 ± 12,506.55 in 2009 to USD 4,345.3 ± 11,104.16 in 2015, while asthma-related costs increased from USD 838.5 ± 3,586.13 to USD 1,772.5 ± 4,960.14 over the period 2009–2015 in Japan (24). Another Japanese study demonstrated a mean annual cost of ¥357,958 (USD 3,113) for managing severe asthma per patient-year (28). A study conducted using the South Korean national database reported direct costs for SA management in 2014, ranging from USD 873 for patients without exacerbations to USD 2,438 for those with at least one exacerbation. Additionally, asthma-related direct costs per patient were estimated at USD174 for level 1, USD 634 for level 2, and USD 1,635 for level 3 SA patients (27). Between 2002 and 2015, SA-related costs rose from USD 2,157 to USD 3,856, outpatient clinic visit costs increased slightly from USD 308 to USD 330, while asthma-related prescription costs decreased from USD 423 to USD 408 during the same period (20).

In Thailand, the healthcare cost for SA patients was USD 658 ± USD 414, with an additional annual cost of USD 71 (29). In Singapore, the mean annual direct asthma cost per person for uncontrolled SA was S$2,952 ± S$4,225 (USD 2,215.65–3,170.97) in 2015 (26). Taiwan reported total all-cause medical costs for SA patients between 2013 and 2016 as USD 8,812.6 and USD 6,517.8, while asthma-related costs were USD 1,562.1 and USD 1,967 for severe eosinophilic asthma (SEA) and high-dose inhaled corticosteroids (HD ICS) SEA, respectively (21). This suggests that SA, with exacerbations and especially in its advanced stages, places a considerable strain on healthcare systems.

##### 3.2.2.2 Loss of productivity

Patients with SA frequently struggle with loss of productivity due to their disease. According to a cross-sectional study conducted between 2010 and 2013 using data from the China National Health and Wellness Surveys (NHWS), absenteeism was 17.84%, presenteeism was 51.73%, and overall work impairment was 58.21%. Under the self-reported definition, absenteeism slightly increased to 18.11%, while presenteeism and overall work impairment were 51.9% and 51.27%, respectively (16).

### 3.3 Mortality

Mortality data on SA in the Asia region is limited. In Japan, between 2013 and 2019, the proportion of all-cause deaths among patients with SA ranged between 10.29% and 11.20% (17). However, in Korea, the asthma-associated death rate increased significantly, rising from 1,917.3 per 100,000 asthmatics in 2003 to 2,650.2 per 100,000 in 2015, according to the study conducted using the National Health and Injury Surveillance System (NHISS) database, suggesting concerns over asthma control (20). The lack of data in other countries underscores the need for comprehensive research on asthma to better understand and address SA-related mortality.

**Table:** 

Severe asthma is associated with greater economic burden, higher rates of hospitalization and healthcare costs, more frequent emergency visits, greater loss of productivity and marginally increased mortality

### 3.4 Challenges in SA management

**Figure 2:**
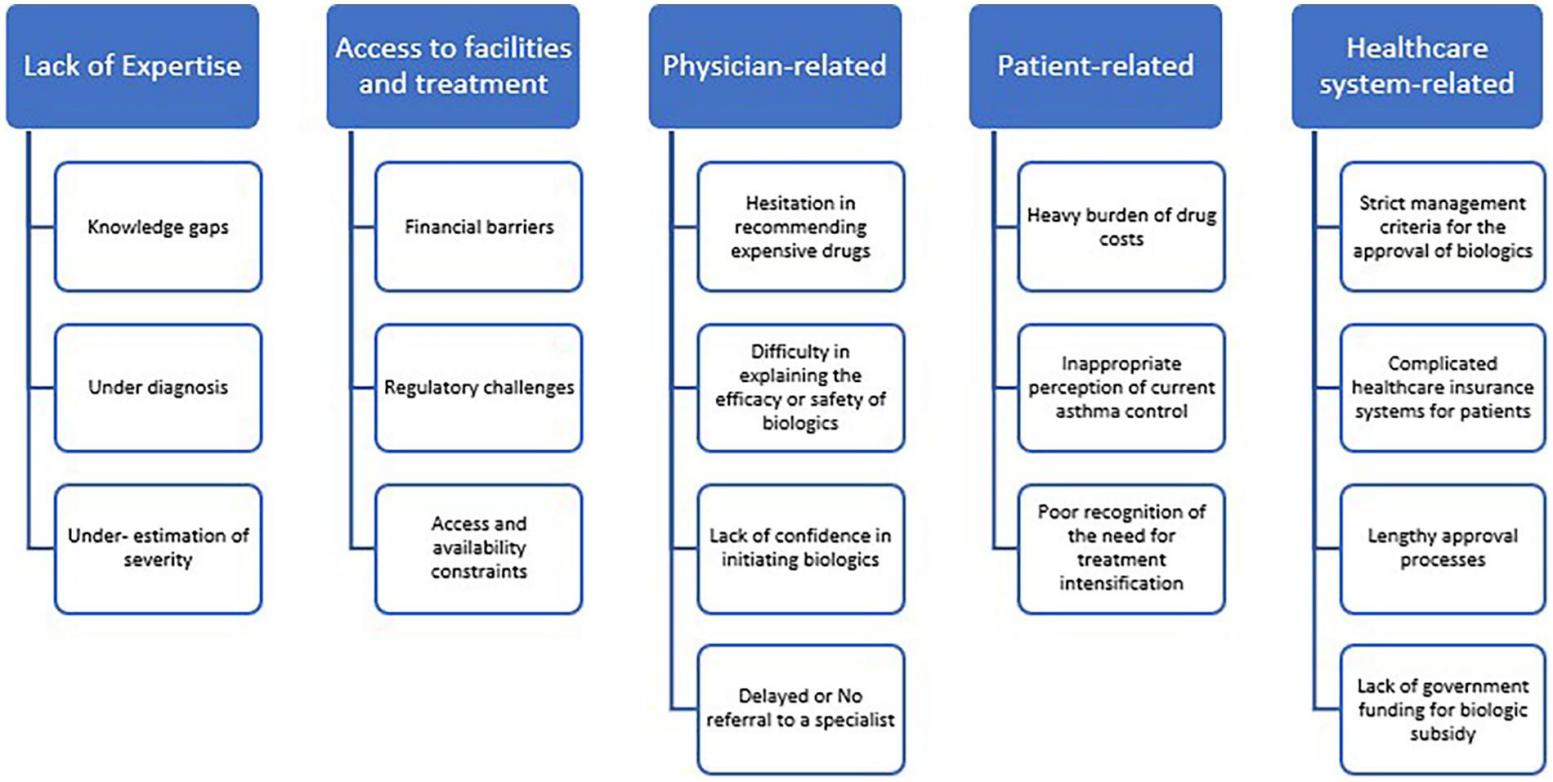
Challenges in severe asthma management.

Figure 2 summarises the most common challenges that pose as barriers to optimal SA management.

#### 3.4.1 Lack of expertise

SA management faces several challenges across Asia, including underdiagnosis, undertreatment, and knowledge gaps. A systematic multidisciplinary approach involving asthma specialists, nurses, pharmacists, and other allied health professionals is desirable, and referral to specialists is warranted in most cases (30, 31). In Japan, there is poor recognition of the need for treatment intensification, and knowledge gaps in diagnosis and treatment still exist (32). Similarly, India faces knowledge gaps and a lack of therapeutic choice selection (33). Both India and Thailand experience issues with improper guidance on inhaler use (30, 33, 34). Underdiagnosis or under-estimation of SA is a widespread challenge in India, Japan, Korea, and other parts of Asia, while under-treatment of SA is prevalent in Japan, Thailand, and across Asia (30–38).

#### 3.4.2 Access to facilities and treatments

Access to biologics is limited across many countries in Asia, mainly due to inadequate reimbursement, limited access, or high costs (39). Financial barriers and inadequate diagnostic infrastructure further exacerbate the issue, making advanced treatments unaffordable for many patients in these regions. Regulatory and approval challenges add to delays in the availability of new therapies (10, 17, 31–33, 35, 40–43). The EVEREST study conducted by Le et al. emphasized regulators' need to enhance access to biologics (44).

#### 3.4.3 Over-reliance on oral corticosteroids

The high burden of oral corticosteroid (OCS) use in SA management is a critical concern across various parts of Asia. Studies conducted in South Korea demonstrated a high prevalence of OCS- dependence of 21.5% amongst the severe asthma patients. The chronic use of OCS is associated with a higher and dose-dependent risk of mortality (44, 45). Further research by Kwon et al. found that OCS users in South Korea exhibited poorer lung function, higher medication usage, and more frequent exacerbations compared to non-users (46). Additionally, OCS use is associated with higher consumption of rescue medication, more frequent emergency room (ER) visits, and a higher incidence of anxiety (47). In Japan, a rise in regular OCS use was observed from 59% to 76%, while only 12% of patients were receiving biologic therapies, highlighting potential barriers such as high treatment costs (17). In India, the overuse of OCS is often attributed to gaps in knowledge and skills among healthcare providers, in both public and private sectors, leading to a preference for oral medications over more advanced treatment options (33). A recent study from the Singapore Severe Asthma Registry revealed a prevalence of maintenance OCS was 17.7% and a very high prevalence of Diabetes Mellitus of 20.1% in the population compared to 3.7% in Austria. Some ethnic differences in potentially OCS- related comorbidities were also observed: Malay and Indian patients were more likely to have obesity (Chinese 17.7%, Malay 50.9%, Indian 40.9% *p* < 0.001); Indian patients were most likely to have diabetes mellitus (Chinese 16.6%, Malay 18.2%, Indian 38.6%, *p* = 0.006)) while Chinese patients were most likely to have osteoporosis (Chinese 14.5%, Malay 0%, Indian 2.3%, *p* = 0.001), underscoring possible ethnic differences in susceptibility to OCS- related complications (48).

#### 3.4.4 Others

Suboptimal referral to specialists is an issue observed in Japan and the Asian region, limiting access to advanced care (31, 32). Patient identification and diagnosis remain problematic, with underestimation of asthma severity across Asia and poor perception of asthma control in Japan and South Korea. Additionally, the association of SA with multiple comorbidities is evident in Japan and Thailand (17, 30, 49).

In terms of lack of research, limited evidence-based treatment options are the major barrier observed in Japan and other parts of Asia (2, 3, 10) with an underrepresentation of Asian patients in Phase II and Phase III clinical trials (50). Patient adherence to treatment is also a challenge, with poor adherence reported in India, Thailand, and across Asia (30, 33, 51).

**Table:** 

Severe Asthma care across the Asian region faces numerous challenges in delayed patient identification or referral, limited access to advanced investigations and treatment, limited expertise, poor patient adherence, and lack of government support and funding, which underscore the need for a more comprehensive approach to severe asthma. In Asia, the higher burden for SA can be attributed to unmet needs such as late diagnosis, suboptimal treatment, limited access to biologics and SA centres, reliance on oral corticosteroids, and corticosteroid-related comorbidities. OCS use in SA management across Asia is associated with high dependence, increased health risks, and significant regional and ethnic disparities, often driven by limited access to advanced therapies and gaps in clinical practice

### 3.5 Phenotypes in Asian severe asthma patients based on inflammatory biomarkers and co-morbidities

#### 3.5.1 Severe asthma phenotype

Severe asthma is classified into distinct phenotypes and endotypes. The phenotypes are classified based on pathophysiology and molecular markers, which guide targeted treatment strategies, particularly for biologics. There are two phenotypes: T2-high (demonstrated by prominent eosinophilic infiltration of the airway mucosa and overexpression of T_H_2-dependent cytokines (IL-4, IL-5, and IL-13) and T2-low (demonstrated by the neutrophilic and pauci-granulocytic airway infiltrates and specific cytokines including IL-17, IL-6, IL-8, IL-22, and epithelial cytokines) (52, 53). The T2-low phenotype includes neutrophilic and paucigranulocytic subtypes and is associated with Th1 and Th17 cell activation, along with elevated IL-17A levels. GINA defines T2-high asthma as having blood eosinophil counts ≥150 cells/μL and/or fractional exhaled nitric oxide (FENO) of ≥20 ppb and/or Sputum eosinophils ≥2% and/or allergen driven asthma with Ig E ≥30 IU/mL and sensitization to any allergen on specific serum IgE or skin prick testing and/or need for maintenance OCS (repeat blood eosinophils and Feno up to three times on lowest possible OCS) (1, 5). However, the definition of T2 high asthma may differ across the literature (54–56).

#### 3.5.2 Predominant phenotypes of severe asthma in Asia

Although the Asian patients do not demonstrate a single unique phenotype, the Type 2 High phenotype is predominant in the region. The prevalence of the T2-high phenotype in severe asthma patients across Asia ranges from 49% to 97.5% (41, 57–62), with eosinophilic asthma ranging from 34% to 76.8% (28, 41, 48, 57, 58, 63, 64), and studies consistently show significant overlap between eosinophilic and IgE-mediated phenotypes, particularly in countries like Singapore, China, India, and Japan (48, 57, 63).

Although asthma phenotypes in Asia are generally similar to those in Western countries, certain clinical differences have been observed. A comparison of severe asthma cohorts—SARP (USA), U-BIOPRED (Europe), ProAR (South America), and COREA (Korea)—revealed notable regional variations. The COREA cohort included older patients (mean age: 50.79 ± 15.75 years) with predominantly late-onset asthma (mean age of onset: 45.74 ± 16.80 years), whereas other cohorts, such as ProAR, had earlier onset (mean age: 14.14 ± 15.36 years). Pollen sensitization was the most common allergen in the SARP group, while house dust mite sensitization was more prevalent in the ProAR, COREA, and Singapore cohorts (65, 66). A study by Kim et al. found that Korean patients with severe asthma were typically older, had been undergoing treatment for about 10 years, and half had a history of smoking (67). These differences may be attributed to delayed asthma diagnosis, airway remodelling with fixed airflow obstruction, the presence of comorbidities, and suboptimal asthma management.

**Table:** 

Similar to the Global International Severe Asthma Registry (ISAR) data but with slight variations, the majority remains T2 high, with differences in proportions depending on the data source or definition used

### 3.6 Definition of clinical remission from an Asian perspective

Clinical remission in severe asthma is defined differently across various studies and guidelines. However, key features considered for the definition of clinical remission include ACT score, exacerbation status, and lung function. Table 1 summarises the prevalent definitions used across studies and guidelines worldwide.

**Table 1:** Various prevalent definitions of clinical remission in asthma.

Clinical Remission	
GINA (1)	Clinical remission is defined as a period of at least 12 months without symptoms [assessed with validated tests like the Asthma Control Test (ACT) and Asthma Control Questionnaire (ACQ)], stabilization or optimization of lung function, patient involvement and agreement about remission, and no use of systemic corticosteroids
ISAR Data (68)	Clinical remission in patients initiated on biologics through two domains that include exacerbation rate and long-term oral corticosteroid (LTOCS), three domains which include exacerbation rate + LTOCS + asthma control or exacerbation rate + LTOCS + percent predicted FEV_1,_ or all four domains that include annualized exacerbation rate, Asthma control, Daily LTOCS dose, and Lung function
PGAM (Practical Guidelines for Asthma Management: for general practitioners in Japan) (69)	an asthma control test (ACT) score ≥ 23 points, absence of exacerbations, and no use of systemic corticosteroids (a lung function criterion was not used in this guideline)
Japanese guidelines for adult asthma 2024 (70)	Clinical remission is defined as: No use of systemic corticosteroids, at least 12 months without symptoms [the Asthma Control Test (ACT) ≥23 points and Asthma Control Questionnaire (ACQ) ≦0.75], absence of exacerbations, and normalization or optimization of lung function
Chinese Guideline for bronchial asthma prevent and management (2024 edition)on Severe Asthma (4)	Clinical remission is defined as After initiating biologic therapy or other anti-asthma therapy, patients achieving ≥ 1 year of symptom-free status, no exacerbation, normal/near-normal lung function, and no oral corticosteroids (OCS) requirement
American College of Allergy, Asthma, and Immunology (ACAAI), American Academy of Allergy, Asthma, and Immunology (AAAAI) and American Thoracic Society (ATS) workgroup (71)	Clinical remission is defined using six criteria: No exacerbations requiring physician visit, emergency care, hospitalization, and/or systemic steroid for asthma, No missed work or school over 12 months due to asthma-related symptoms, stable and optimized pulmonary function results on all occasions measured over a 12-month period with a minimum of two measurements during the year, Treatment with ICS only at low-medium doses (or less), an ACT score >20 (or an AirQ score <2 or an ACQ score <0.75) on all occasions measured in the previous 12-month period with a minimum of two measurements during the year, Symptoms requiring one-time reliever therapy no more than once a month
Global study (SIROCCO, CALIMA, and ZONDA trials) (72)	Achieving zero exacerbations, zero oral corticosteroids (OCS) use, asthma control questionnaire (ACQ-6) ≤0.75, and pre-bronchodilator (pre-BD) FEV_1_ increase ≥100 mL
German Respiratory Society S2k Guidelines (71, 73)	Criteria for asthma remission—all criteria must be Sustained (≥12 months) absence of asthma symptoms Sustained (≥12 months) absence of exacerbations Stable lung function No need for systemic glucocorticoids for the treatment of asthma
REDES study (Spain) (74)	Four-component criteria: (i) OCS-free; (ii) exacerbation-free (for 52 weeks); (iii) an ACT score ≥20; and (iv) a percent predicted post-bronchodilator FEV_1_ ≥80%. Secondly, a three-component clinical remission definition that included meeting the following criteria at Week 52: (i) OCS-free; (ii) exacerbation-free (for 52 weeks); and (iii) an ACT score ≥20
DESTINATION study (Global study includes various Asian territories) (75)	Asthma Control Questionnaire-6 score ≤1.5, stable lung function (a forced expiratory volume in 1 s >95% of baseline) at the end of each year, and no exacerbations or use of oral corticosteroids during the time periods assessed
SANI registry (76)	Partial clinical remission when there was no need for OCS and two of three criteria were met: the absence of symptoms, the absence of exacerbations or attacks, and the stability of lung function for at least 12 months with ACT score of 20/25 to 25/25 and Asthma Control Questionnaire score of <1 Complete clinical remission when there is no further need to use oral corticosteroids and all three criteria are met

Considering the distinct clinical characteristics discussed earlier, the definition of clinical remission should be re-evaluated with a focus on Asian patients. While all prevalent definitions concur on the criteria related to exacerbations, use of oral corticosteroids, and symptoms, a notable difference in lung function criteria is evident. It may not be possible for the patient to achieve optimal lung function as defined by the prevailing definitions, considering the possibility of existing airway remodelling (77). The experts opined that a more realistic and achievable target may be beneficial instead of the pre-set criteria currently utilised in other geographies.

**Figure 3:**
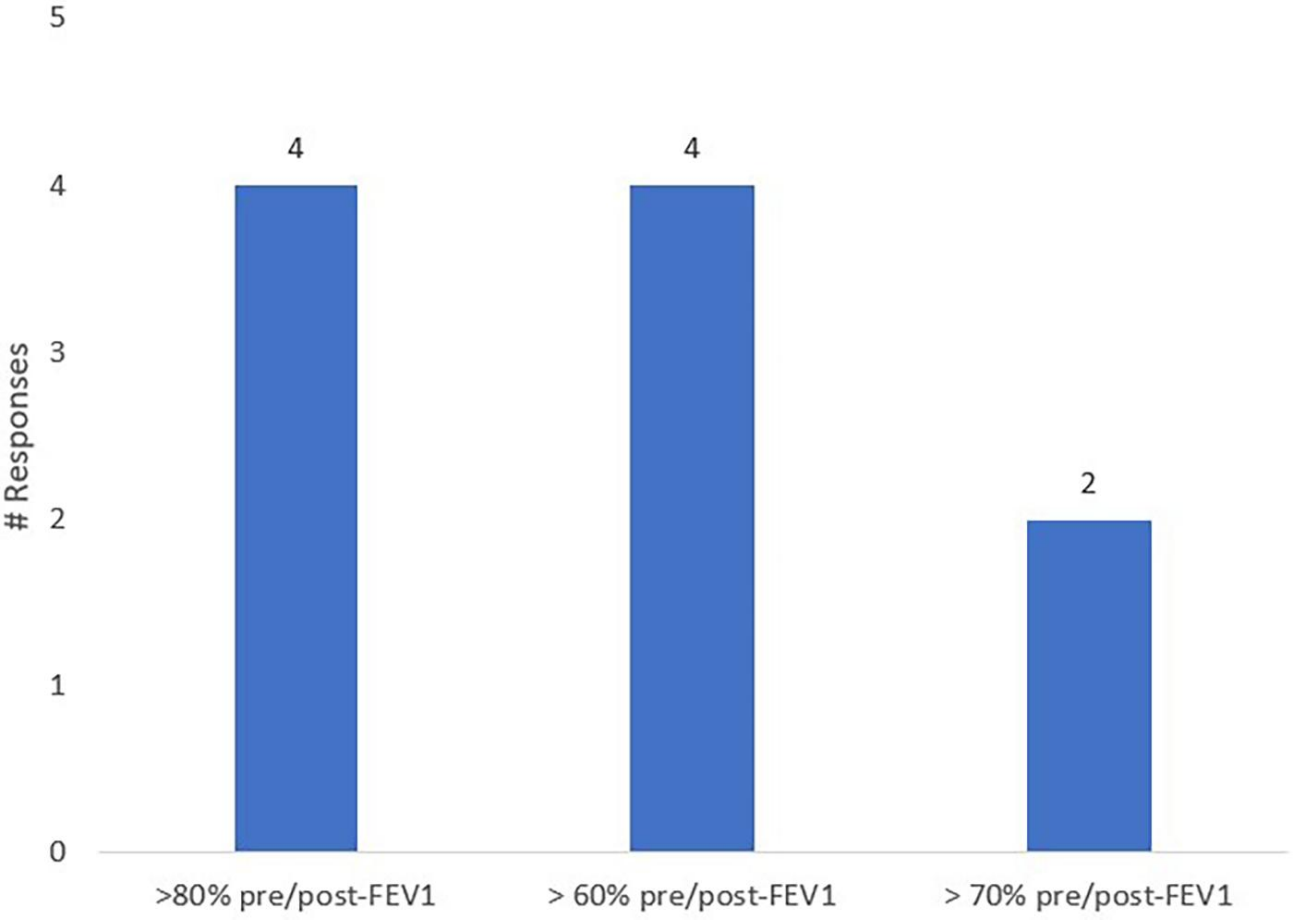
Survey: expert opinion for lung function criterion to define clinical remission in Asia.

In order to understand the lung function criterion for defining clinical remission, the experts in the panel answered a survey (Figure 3). In the survey, most experts suggested that the lung function criterion should be incorporated into the definition of clinical remission, alongside the other three criteria: Exacerbations, OCS use, and symptoms. Additionally, experts suggested that the lung function criterion should encompass either the stabilization of lung function alone or both stabilization and normalization. They also proposed that a lack of bronchodilator response and declined lung function could be included in the lung function criterion, and regular monitoring of Peak Expiratory Flow Rate (PEFR) could provide insight into daily lung function changes, especially in patients with asthma. Regarding the optimal value of pre- or post-bronchodilator FEV_1_ for a patient to be classified as being in clinical remission, experts had diverse opinions, with a few mentioning >80% pre/post-FEV_1_, others mentioning >60% pre/post-FEV_1_, and some mentioning >70% pre/post-FEV_1_. Experts also suggested that, along with FEV_1_ value, the presence or absence of symptoms such as cough or dyspnoea should be considered. In light of the inadequate data currently available to achieve an Asia-specific definition for clinical remission, further studies in the region are desirable. The experts also highlighted having a robust lung function criterion, considering the evolving treatment landscape with the availability of biologics, which may make the lung function targets achievable in the future.

**Table:** 

Proposed definition of clinical remission based on four criteria:
1. Sustained good control of asthma symptoms for a minimum of 12 months
2. Sustained absence of exacerbations for a minimum of 12 months
3. Free from use of OCS
4. Pre/post-FEV_1_ ≥80% predicted OR attainment of best personal lung function at a stable state with the use of the currently available therapeutic agents including biologics

### 3.7 Co-morbidities impacting SA management

The presence of various co-morbidities complicates the management of SA (Table 2).

**Table 2:** Common comorbidities associated with SA.

Comorbidity Type	Specific Comorbidities	Prevalence	References
Metabolic/Lifestyle-related	Obesity, Diabetes Mellitus (DM), Hypertension, Dyslipidaemia	Frequently co-occur; associated with poorer asthma control	(17, 41, 56, 61, 78–85)
Psychological	Depression, Anxiety, Mood disorders	Common in severe asthma; may be steroid-related	(17, 41, 56, 61, 78–85)
Allergic/Type 2 Inflammation	Allergic rhinitis, Chronic sinusitis, Atopic dermatitis, Nasal polyps, Urticaria, Anaphylaxis	Strongly linked with type 2 inflammation	(17, 41, 56, 61, 78–86)
ENT/Respiratory-specific	Otitis media, Sleep apnoea, Allergic bronchopulmonary mycosis (ABPM)	ENT-related comorbidities common; ABPM specific to asthma	(17, 41, 56, 61, 78–85)
Chronic Cough Conditions	COPD, GERD, CHF	Contribute to chronic cough and asthma exacerbations	(17, 41, 56, 61, 78–85)
Chronic Infectious Diseases	Nontuberculous mycobacteria (NTM), Tuberculosis, Chronic pulmonary aspergillosis (CPA)	Frequently observed in severe asthma patients	(17, 41, 56, 61, 78–85)
Steroid-related Complications	Osteoporosis, Adrenal suppression, Mood disorders	Due to long-term corticosteroid use	(17, 41, 56, 61, 78–85)
Rare Eosinophilic Disorders	Eosinophilic pneumonia, Eosinophilic granulomatosis with polyangiitis (EGPA)	Less common but impactful	(17, 41, 56, 61, 78–85)
Country-specific Data	Perennial allergic rhinitis (India), Rhinosinusitis (Thailand)	India: ↑ prevalence with asthma severity; Thailand: 54% in SA cases	(30, 86)

### 3.8 “Treat-to-target” approach via achieving clinical remission as the major treatment goal in severe asthma\

The Treat-to-Target approach in asthma is a management strategy that involves systematically assessing specific characteristics across respiratory, extra-respiratory, and behavioural domains and focuses on achieving and maintaining clinical remission by setting individualized goals and tailoring therapy accordingly to reach and maintain those targets (87, 88). Adopting a treat-to-target approach can achieve clinical remission in severe asthma. Precision medicine represents a promising way, marking a shift from “treat-to-failure” to “treat-to-target,” with clinical remission as the ultimate goal for SA management (72). In the SHAMAL study, 92% of patients successfully reduced their high-dose ICS, with over 60% transitioning to using only anti-inflammatory relievers without any loss of asthma control. Despite significant reductions in ICS, more than 87% of patients in the treatment-reduction group remained free from exacerbations by week 48. Additionally, over half of the patients who reduced their background medications achieved clinical remission by week 48. There was a decline in FEV_1_ in some patients, reducing their ICS as-needed only (89). Considering these findings, GINA recommends stopping other add-on medications, especially OCS, and reducing the ICS-LABA dose in SA patients on biologic therapy plus high-dose ICS-LABA who show a good response to the treatment. However, it also recommends that the ICS-LABA dose should not be completely stopped (1).

In addition to clinical remission as a target, additional targets such as eosinophils, lung function, and type-2 inflammation are also being increasingly considered as targets for treatment (90–92).

**Table:** 

Clinical remission is attainable, and this concept is supported by GINA. A treat-to-target approach is recommended, with the next steps including tapering background medications.
In addition to clinical remission as a target, eosinophils and other inflammatory markers appear to be promising targets for asthma management

### 3.9 Biologics and their impact on the Asian severe asthma population

Various biologics, including anti—Ig E (Omalizumab), IL-5 inhibitors (mepolizumab and reslizumab), IL-5 receptor inhibitors (benralizumab), anti-IL-4R*α* inhibitors (dupilumab), and anti-thymic stromal lymphopoietin (Tezepelumab), have become available in many Asian countries. Similar to Western populations, these biologics have shown comparable effectiveness among Asian patients. Studies conducted in Thailand (93), South Korea (94, 95) and China (4) highlighted that omalizumab reduces asthma exacerbations, hospitalization rates, and the use of OCS while enhancing asthma symptom control, quality of life, lung function, and reducing airway remodelling.

Similarly, benralizumab has been shown to decrease peripheral blood eosinophils, acute asthma attacks, and OCS use, with improvements in lung function and symptom control (96). The MIRACLE study conducted in Asia demonstrated that benralizumab significantly reduced annual exacerbation rates and improved lung function, asthma symptoms, and health-related quality of life (97). The TOAST study, a prospective real-world trial in Japan, also confirmed the effectiveness of benralizumab in improving asthma control, quality of life, and lung function while reducing OCS use; higher baseline eosinophil counts (≥400/μL) and FeNO levels (≥22 ppb) were associated with better response (98).

In the *post hoc* analysis of the Korean cohort from the DREAM and MENSA studies, mepolizumab improved lung function and quality of life in patients with severe eosinophilic asthma (99).

Dupilumab has also proven effective in reducing the risk of acute asthma attacks and improving asthma control, quality of life, and lung function while reducing the OCS dependency. Additionally, in the Korean subgroup of the LIBERTY ASTHMA TRAVERSE study, dupilumab was associated with improvements in lung function and exacerbation rates (100).

Data from the KoSAR Biologics Registry further corroborate the benefits of these biologics, demonstrating significant improvements in asthma control and patient quality of life (35). ISAR studies, which include 23 countries (from the APAC region: India, Malaysia, Japan, Korea, Singapore, Taiwan) and 323 patients from the APAC region, further highlight the benefits of biologics in patients. Patients treated with anti-IgE, anti-IL5/5R, and anti-IL4Rα achieved four-domain remission within a year of initiation, with greater chances of remission seen in those with less severe impairment and shorter asthma duration (68). Anti-IgE and anti-IL5/5R were effective in reducing exacerbations, hospitalizations, and long-term oral corticosteroids (LTOCS); however, anti-IL5/5R was more effective when compared with a generally improving anti-IgE (101).

A meta-analysis by Kyriakopoulos et al, demonstrated that Anti-IL4α (dupilumab) and anti-Thymic stromal lymphopoietin (TSLP) (Tezepelumab) were the most effective in reducing exacerbations and improving FEV_1_. Anti-TSLP was particularly effective in reducing hospitalizations. Anti-IgE (Omalizumab) notably reduced the ACQ score and improved the AQLQ score. Anti-IL5/5Rα agents (Mepolizumab, Reslizumab, and Benralizumab) were effective in reducing OCS use and facilitating discontinuation (102).

Experts concurred on the efficacy of biologics but highlighted the key limiting factors for greater utilisation are limited access and lack of reimbursement of biologics in the Asia-Pacific region. In order to achieve the treatment targets and select the appropriate biologic for the right patient, it is important to have all the possible biologics available.

Tables 3, 4 demonstrate the availability and reimbursement status of Biologics in Asian countries as mentioned by the experts as of January 2025.

**Table:** 

• Identifying appropriate patients for biologic treatment through phenotyping and initiating timely and suitable interventions can improve outcomes and reduce emergency room visits and hospitalizations for those with severe asthma (SA).
• All classes of biologics should be made available in Asia for appropriate biologic selection.

**Table 3:** Availability of biologics across different Asia-Pacific territories.

Asia Pacific Territories	Availability					
	Omalizumab	Benralizumab	Dupilumab	Mepolizumab	Tezepelumab	Reslizumab
China	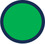	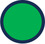	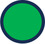	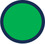	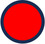	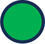
Hong Kong SAR	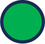	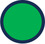	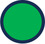	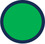	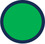	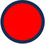
Japan	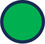	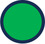	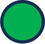	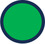	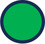	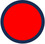
Korea	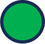	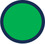	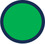	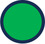	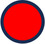	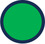
Malaysia	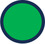	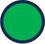	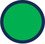	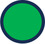	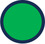	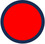
Singapore	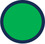	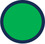	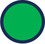	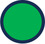	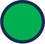	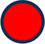
Taiwan	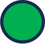	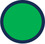	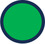	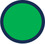	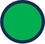	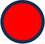
Thailand	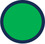	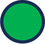	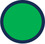	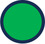	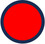	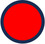
Vietnam	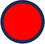	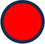	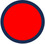	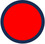	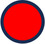	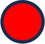
India	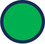	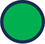	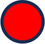	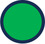	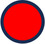	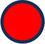

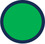
, Available; 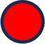, not available; SAR: special administrative region.

**Table 4:** Government reimbursement status of biologics across different Asia-Pacific territories.

Asia Pacific Territories	Reimbursement Status					
	Omalizumab	Benralizumab	Dupilumab	Mepolizumab	Tezepelumab	Reslizumab
China	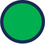	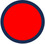	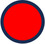	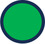	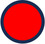	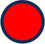
Hong Kong SAR	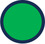	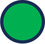	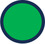	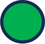	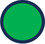	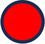
Japan	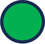	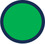	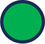	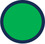	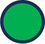	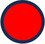
Korea	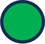	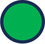	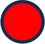	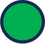	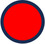	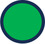
Malaysia	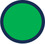	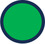	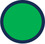	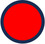	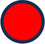	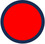
Singapore	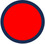	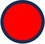	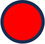	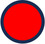	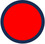	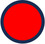
Taiwan	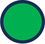	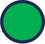	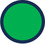	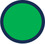	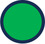	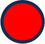
Thailand	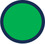	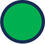	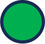	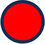	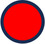	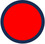
Vietnam	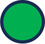	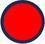	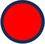	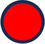	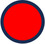	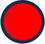
India	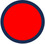	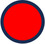	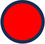	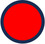	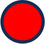	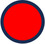

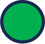
, Available; 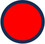, not available; SAR: special administrative region.

Identifying appropriate patients for biologic treatment through phenotyping and initiating timely and suitable interventions can improve outcomes and reduce emergency room visits and hospitalizations for those with severe asthma (SA).

All classes of biologics should be made available in Asia for appropriate biologic selection.

### 3.10 Selection of the right biologic for the right patient

**Figure 4:**
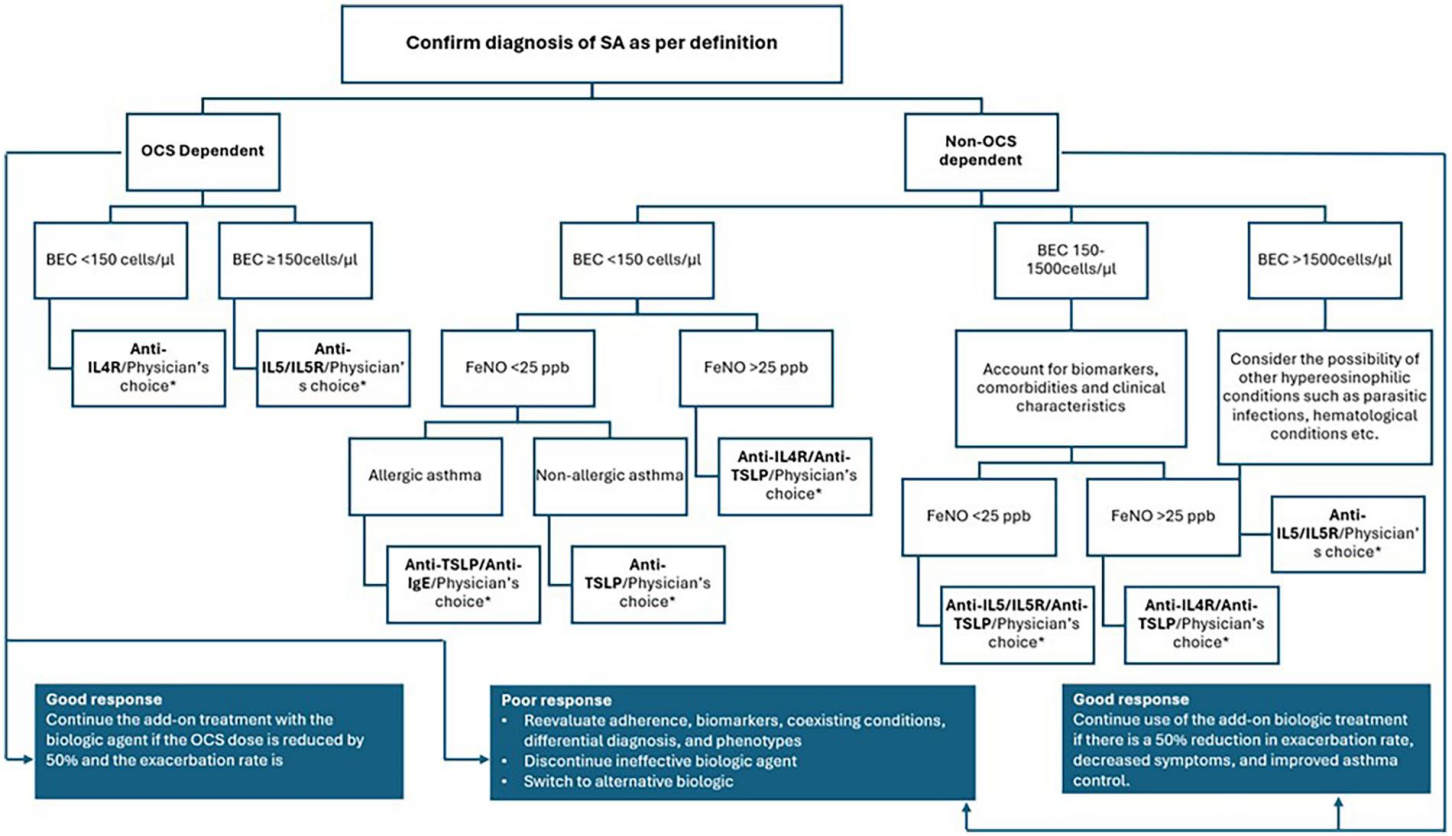
Treatment algorithm for biologic selection in patients with severe asthma.

As summarized by Brusselle et al., there are no direct comparisons from randomized controlled trials (RCTs) on the effectiveness and safety of monoclonal antibodies for severe asthma; there is a lack of strong evidence to guide treatment choices. Before starting biologic therapy, it is important to record the number of asthma attacks in the past year, use of OCS, biomarkers (like blood eosinophil count, FeNO, and specific IgE levels), lung function (FEV_1_), asthma control, and quality of life. When selecting a therapy, several factors should be considered, including biomarkers, comorbidities (such as eczema and nasal polyps), dosing frequency and schedule, route of administration, the need for healthcare staff monitoring, the age of asthma onset, as well as the availability and affordability of the biologic. Insurance coverage, cost, and patient preferences are also important factors. These factors, along with clinical characteristics, guide the choice of the initial biologic therapy (1, 103) The expert panel recommended assessing coexisting allergic or immunologic conditions (e.g., chronic rhinosinusitis, urticaria), comorbidities, and the specific treatment indication before starting therapy. A possible treatment algorithm considering the currently available classes of biologics and based on the concept presented by Brusselle et al. (103) is summarised in Figure 4. However, it should be noted that the utility of this algorithm may be considered limited, considering the limitations on availability and access to these treatments in Asian countries.

**Table:** 

Biologics have been shown to reduce exacerbations, reduce ER visits or hospitalizations, reduce OCS use, and associated OCS complications in patients with SA

## 4 Discussion

Call for actions in improving the standard of severe asthma care in Asia.

### 4.1 Adoption of biologics and other life-changing therapies

Prioritizing individualized care and actively involving patients in decisions around monoclonal antibody therapies for severe asthma is essential. Patient preferences should be respected when initiating biologics, selecting specific agents, and defining treatment goals. In India, this patient-centred approach is gaining increasing recognition (40). Similarly, Japan has called for early intervention, enhanced education on biologics, and flexibility in switching treatments as needed. Improved communication with patients, especially those facing income constraints, is vital, alongside more lenient approval criteria for biologics. Policymakers and governments must take steps to ease the financial approval process for biologics, ensuring that patients in need have better access to these treatments.

There is a clear need for evidence-based validation of biologics and development of novel treatments beyond traditional treatments (32). Liano et al. demonstrated that only a small proportion of patients on biologics achieve remission, underscoring the potential need for therapy switches, combination therapies, or earlier interventions to improve remission rates, using ISAR data (68). Le et al. further highlighted the persistent high disease burden among patients with severe asthma who lack access to T2-targeted biologics, emphasizing the critical unmet medical need. This underscores the importance of regulators increasing and standardizing access to biologics and researchers developing more effective treatment options for better asthma management (44).

Recent data from ISAR demonstrated that biologics can prevent several OCS-related comorbidities, including diabetes mellitus, major cardiovascular events, and anxiety/depression, highlighting the need for early access and treatment of SA before irreversible damage and harm are done (104).

In many LMICs, high costs, insufficient resources, lack of proper asthma management tools, limited physician expertise, and patients' financial constraints further hinder the availability and effective use of biologics (105). According to Swarnakar et al., though certain biologics are available through India's public healthcare system, the high costs of these treatments in private markets remain a barrier to broader access (33).

To improve the management of severe asthma, advocacy for equitable access to biologic therapies, earlier intervention, and greater flexibility in treatment options is imperative. Policymakers and governments must ease the approval criteria for biologics, reducing financial barriers and ensuring access to life-changing treatments.

### 4.2 OCS stewardship

Implementing OCS stewardship is key to improving the management of SA in Korea by addressing the risks associated with frequent OCS use and ensuring patient awareness (106). Japan similarly prioritizes reducing systemic corticosteroid dependence among SA patients and highlights the need to overcome barriers to biologic adoption, especially due to high costs and low uptake among OCS-dependent patients (17).

### 4.3 Diagnosis and Biomarker phenotyping

In China, there is a strong emphasis on advancing research into the clinical phenotypes and endotypes of severe asthma. This includes a focus on developing biomarkers accurately, completely, and repeatedly to improve diagnosis and classification (4).

### 4.4 Referral network

In Korea, a strong referral system from primary care to asthma specialists, supported by partnerships between general practitioners and asthma clinics and reinforced through education initiatives, is advocated to enhance referral rates (35). In Japan, interprofessional cooperation among doctors, nurses, and pharmacists is emphasized to support patient adherence to long-term asthma management, fostering a more coordinated and effective care approach (107).

### 4.5 Policy shaping and improving access

Venkitakrishnan et al. propose that shared decision-making between patients and physicians may help align treatment goals effectively (40), while increasing insurance coverage, negotiating lower drug prices, and funding research into affordable options can improve access (33). Kim et al. suggest the establishment of specialty severe asthma clinics that include multidisciplinary teams to aid in offering several services such as smoking cessation support, patient education, and inhaler use correction. Expanded insurance reimbursement for eligible patients and a streamlined approval process are also recommended (35). Consensus statements from Chinese experts recommend collaborative research on severe asthma to better understand its clinical and socioeconomic impact. Additionally, evidence-based, individualized therapy is suggested in order to improve quality of life and lower the disease burden (4).

The following strategies are proposed to improve the accessibility and affordability of biologics in the Asia-Pacific region:

Tiered Pricing: Tiered pricing (selling drugs and vaccines in developing countries at prices systematically lower than in industrialized countries) allows pharmaceutical companies to set different prices for the same medication based on the patient's ability to pay or the economic status of different regions. However, tiered pricing should only be used as a short-term measure, applied in cases of limited market volume or production capacity, while planning for long-term affordability. Promoting competition by encouraging multi-source production and generic entry is critical to achieving the lowest sustainable prices. Additionally, avoiding arbitrary market segmentation through fair pricing policies can prevent disproportionately high costs for middle-income countries. This makes essential drugs more affordable for low-income patients (108).
Patient Support Programs (PSPs): Offer financial aid, medication adherence support, disease education, and help with insurance and reimbursement processes, improving overall health outcomes (109).
Patient Assistance Programs (PAPs): Provide free or reduced-cost medications to those who cannot afford them, ensuring access regardless of financial circumstances (109).

### 4.6 Others

In China, there is a call for collaborative research to establish baseline data on severe asthma, identify risk factors, and develop individualized, evidence-based treatment plans. Key research focus areas include type 2 inflammation-targeted biologics, maintenance strategies, and long-term treatment effectiveness to improve asthma control and patient quality of life (4).

In many LMICs across Asia, a lack of data and resources hinders the advancement of severe asthma care. The establishment of specialized asthma and allergy care facilities in these areas would aid in bridging gaps in diagnosis and care (10).

Call for Action

**Table:** 

**R**—Timely Referral
**E**—Empower patients
**A**—Advocate, improve Accessibility to SA centres and Affordability of Biologics
**C**—Collaborate to shape Policy
**H**—reduce Harms of OCS

## 5 Conclusion

The burden of severe asthma (SA) in Asia is substantial, with prevalence rates that may be higher than those in Western countries due to specific regional risk factors such as outdoor and indoor pollution, patient and healthcare system factors. Despite advancements in asthma care, many patients with SA continue to experience uncontrolled symptoms, frequent exacerbations, and reduced quality of life. Key management challenges include inadequate expertise, delayed diagnosis, limited access to treatments like biologics, and reliance on oral corticosteroids.

To address these issues, a panel of Asian respiratory specialists proposed that clinical remission in SA should include symptom control, no exacerbations, no oral corticosteroid use, and stable or normal lung function for at least 12 months. They recommended several key points as a call for action to reduce the overall burden of SA in Asia, including timely referral to SA specialists, accessibility of SA centres of excellence patient empowerment, and advocacy for accessibility and affordability are essential to improve severe asthma care. Collaboration among clinicians and policymakers can shape effective policies, while reducing harms from oral corticosteroids through steroid-sparing strategies ensures safer outcomes. Together, these actions create a comprehensive, patient-centered approach. A comprehensive, multidisciplinary approach is needed to address diagnostic delays, improve clinician expertise, enhance patient adherence, and expand access to advanced therapies.

Increased representation of Asian patients in clinical trials and robust real-world evidence generation are critical for tailoring treatment guidelines to regional needs.

## Glossary

Anti-IgE: anti–immunoglobulin E
Anti-IL4R: anti–interleukin-4 receptor
Anti-IL5/IL5R: anti–interleukin-5 or interleukin-5 receptor
Anti-TSLP: anti–thymic stromal lymphopoietin
BEC: blood eosinophil count
COPD: chronic obstructive pulmonary disease
CRSwNP: chronic rhinosinusitis with nasal polyps
EGPA: eosinophilic granulomatosis with polyangiitis
FeNO: fractional exhaled nitric oxide
HES: hypereosinophilic syndrome
IgE: immunoglobulin E
IL-4R: interleukin-4 receptor
IL-5: interleukin-5
IL-5R: interleukin-5 Receptor
OCS: oral corticosteroids
TSLP: thymic stromal lymphopoietin.
